# Return to sports after arthroscopic debridement and bone marrow stimulation of osteochondral talar defects: a 5- to 24-year follow-up study

**DOI:** 10.1007/s00167-016-3992-6

**Published:** 2016-02-04

**Authors:** I. C. M. van Eekeren, C. J. A. van Bergen, I. N. Sierevelt, M. L. Reilingh, C. N. van Dijk

**Affiliations:** Department of Orthopaedic Surgery, Academic Medical Centre, University of Amsterdam, PO Box 22660, 1100 DD Amsterdam, The Netherlands; Department of Orthopaedic Surgery, Slotervaart Ziekenhuis, Amsterdam, The Netherlands

**Keywords:** Osteochondral defect, Arthroscopic debridement and bone marrow stimulation, Sports resumption, Long-term follow-up

## Abstract

**Purpose:**

Osteochondral defects (OCD) often have a severe impact on the quality of life due to deep ankle pain during and after weight bearing, which prevents young patients from leading an active life. Arthroscopic debridement and bone marrow stimulation are currently the gold standard treatment. The purpose of this study was to evaluate the number of patients that resume and maintain sports to their pre-injury activity level after arthroscopic debridement and bone marrow stimulation.

**Methods:**

This retrospective study evaluated patients treated with arthroscopic debridement and bone marrow stimulation between 1989 and 2008. All patients who were participating in sports before injury were included. The Ankle Activity Scale (AAS) was used to determine activity levels during specific time points (before injury, before operation, after operation and at the time of final follow-up).

**Results:**

Ninety-three patients were included. Fifty-seven (76 %) patients continued participating in sports at final follow-up. The median AAS before injury of 8 (range 3–10) significantly decreased to 4 (range 2–10) at final follow-up.

**Conclusion:**

It is shown that 76 % of the patients were able to return to sports at long-term follow-up after arthroscopic debridement and bone marrow stimulation of talar OCDs. The activity level decreased at long-term follow-up and never reached the level of that before injury. The data of our study can be of importance to inform future patients on expectations after debridement and bone marrow stimulation of a talar OCD.

**Level of evidence:**

Retrospective case series, Level IV.

## Introduction

Osteochondral talar defects (OCDs) are common after an ankle distortion [20]. Approximately 50 % of ankle sprains and fractures are associated with an OCD [10, 16]. OCDs can have a severe impact on the quality of life due to deep ankle pain during and after weight bearing [17]. If these defects are left untreated, patients are unable to live an active life [8, 20]. Surgical treatment is indicated when primary non-operative treatment fails or is insufficient [13]. The primary surgical treatment for OCDs up to 15 mm consists of arthroscopic debridement and bone marrow stimulation with an overall success rate of 85 %, lasting over the years to have a 76 % satisfactory outcome at long term [14, 15, 19]. Success rate is related to pain levels and the ability to perform the activities of daily life; however, these do not take into account the sport activity level of patients. For athletes with an OCD, the lapse before resuming high-impact sport after surgery can be as much as 3–6 months [2, 5, 10]. Recently, a prospective study showed that 80 % of the patients return to their pre-injured sport level within 1 year after arthroscopic debridement and microfracturing of an OCD of the talus [7]. However, little is known about the long-term follow-up of this treatment. The hypothesis was that patients were able to continue sports activity after debridement and bone marrow stimulation in the long term.

The purpose of this study was to evaluate: (1) the proportion of patients that return to sports after arthroscopic debridement and bone marrow stimulation of OCDs at long-term follow-up, and (2) the activity level before injury, before surgery and at long-term follow-up.

## Materials and methods

In a retrospective study, questionnaires were used to evaluate patients treated at our department with arthroscopic debridement and bone marrow stimulation of an OCD of the talus between March 1989 and December 2008. All patients who participated in sports before injury were included. The exclusion criterion was a concomitant OCD of the tibial plafond.

The Ankle Activity Scale (AAS) by Halasi et al. was used to determine sporting and activity levels at specific time points (before injury, before operation, after operation and at the time of final follow-up). The AAS is a single-page survey system that contains 53 sports, 3 working activities and 4 general activities. Zero points indicate the lowest activity and 10 points indicate the highest activity [3]. The AAS is a specific tool to evaluate sports activity for the ankle joint based on the Tegner score. Reliability for the AAS is not yet reported; however, reliability of the Tegner score for knee-specific injuries is accepted [1].

Secondary outcomes were clinical history, body mass index (BMI), numeric rating scale (NRS) and Foot and Ankle Outcome Score (FAOS) [9, 12, 18]. The reliability for the FAOS is accepted with an ICC of the subscales ranging from 0.83 to 0.88 and a small detective change of 2.1 and 2.5 [12]. Furthermore, patients rated their overall satisfaction with the operation (1 = poor, 2 = average, 3 = fair, 4 = good, 5 = excellent) and indicated whether the complaints had changed since the operation (0 = extremely deteriorated, 1 = significantly deteriorated, 2 = some deteriorated, 3 = unchanged, 4 = little improved, 5 = significantly improved, 6 = completely restored).

In accordance with the ethical standards as were laid down in the 1964 Declaration of Helsinki, approval of an ethics committee for the questionnaires was not required, as confirmed by the ethics committee of our institution (Amsterdam Medical Centre; reference number W15-251#15.0296).

### Statistical analysis

SPSS statistics 22 (Chicago, IL) was used for statistical analysis. Descriptive statistics of continuous variables were calculated as means ± standard deviations or median and ranges in case of a skewed distribution. Categorical variables were presented as frequencies with percentages. Due to skewed distributions and ordinal character of the outcome variables, analysis was performed nonparametrically. Continuous outcome variables (AAS, NRS, FAOS) and categorical baseline variables (sex, side, location and primary of secondary defect) were assessed using Mann–Whitney *U* tests or Kruskal–Wallis tests where appropriate. For continuous baseline values, Spearman’s rho was calculated (age at time of surgery and present and BMI). Overall significance of the AAS between different time moments was checked by use of the Friedman’s test. In case of significance, post hoc pairwise comparison of the AAS several time points was performed by use of Wilcoxon signed-rank test. Sports activity was compared at different time points by use of McNemar tests. To compare patients with a longer follow-up to a shorter follow-up, the group was divided into four different follow-up groups (5–9, 10–14, 15–19 and 20–24 years). The level of significance was set at *P* < 0.05.

## Results

**Fig. 1:**
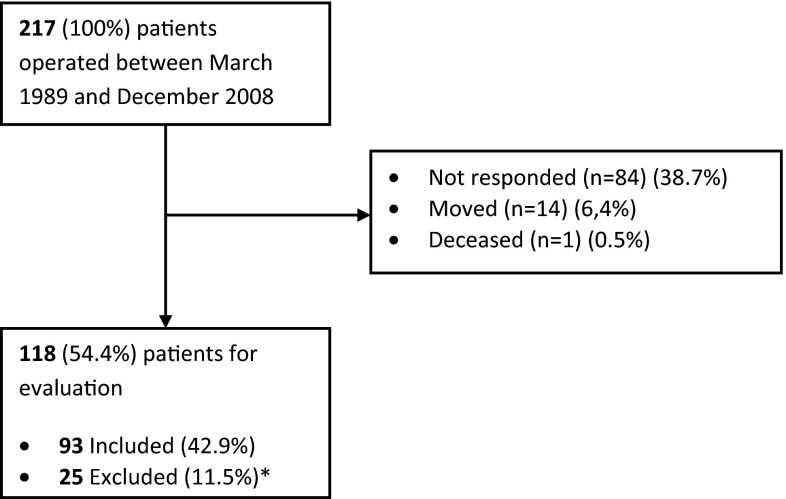
Flowchart. Asterisk Twenty-five patients excluded for not participating in sports before injury

During the study period, 217 patients were available for evaluation of which 118 patients (54.4 %) responded. Twenty-five patients were excluded because they were not participating in sports before injury. Therefore, 93 patients (42.9 %) were evaluated (Fig. 1). The median post-operative follow-up was 118 months (range 46–271; Table 1). Patient’s demographics are displayed in Table 1.

**Table 1:** Patient demographics

Age at time of surgery, mean (SD)	32.6 (9.5)
Age at present, mean (SD)	43.8 (10.7)
Follow-up (months), median (range)	118 (46–271)
Sex, *n* (%)	
Male	61 (66 %)
Female	32 (34 %)
BMI at time of surgery, mean (SD)	25.5 (3.0)
Side, *n* (%)	
Right	55 (60 %)
Left	38 (40 %)
Location, *n* (%)	
Medial	63 (68 %)
Lateral	26 (28 %)
Unknown	4 (4 %)
Defect, *n* (%)	
Primary	68 (73 %)
Secondary	25 (27 %)

Of all 93 (100 %) patients that participated in sports before injury, 57 (61 %) patients participated in a sport before the operation, 72 (77 %) after the operation and 71 (76 %) at the time of final follow-up (Table 2). Significantly fewer patients participated in sports before the operation, after operation and at the time of final follow-up, as compared to pre-injury (*P* < 0.01). The median AAS before injury was 8 (range 3–10), before operation it was 5 (range 2–9), after operation, it was 5 (1–10), and the AAS at time of final follow-up was 4 (2–10). The AAS was significantly higher before injury compared to the other three time points (*P* < 0.01). The AAS did not differ between the different follow-up groups. Moreover, no significant differences were observed in the AAS between follow-up groups at final follow-up. Baseline variables were not significantly associated with the AAS at any time point.

**Table 2:** Number of patients participating in sports and their activity level (AAS)

	Follow-up								Total	
	5–9 years (*n* = 42)		10–14 years (*n* = 34)		15–19 years (*n* = 13)		20–24 years (*n* = 4)			
	Sports participation (%)	AAS	Sports participation (%)	AAS	Sports participation (%)	AAS	Sports participation (%)	AAS	Sports participation (%)	AAS
Before injury	100	9 (3–10)	100	8 (3–10)	100	9 (4–9)	100	7.5 (7–9)	100	8 (3–10)
Before operation	62*	5 (1–10)*	65*	6 (1–10)*	62	4 (1–10)*	50	1 (1–7)	61*	5 (2–9)*
After operation	67*	5 (2–10)*	88^†^	5 (2–9)*	92	6 (2–10)*	50	4 (2–8)	77*	5 (1–10)*
At the time of final follow-up	67*	5 (2–9)*	91^†^	5 (2–9)*	77 %	4 (2–7)*	50	4 (2–7)	76*	4 (2–10)*^‡^
*P* value		n.s.		*P* = 0.01		*P* = 0.01		n.s.		*P* = 0.00

*AAS* Ankle Activity Scale, given in median with ranges

* *P* < 0.05 compared to before injury level; ^†^ *P* < 0.05 compared to before operation; ^‡^ *P* < 0.05 compared to after operation. Overall significance assessed with Friedman’s tests. In case of significance, post hoc pairwise comparison was assessed with the Wilcoxon signed-rank test (for AAS) or by use of McNemar tests (sports participation)

The type of sports often changed during follow-up, as compared to before injury (Table 3). At the time of final follow-up, more patients participated in low-impact sports such as cycling and swimming than before injury.

**Table 3:** Sports participation at different time points

Sport	Before injury (%)	Before operation (%)	After operation (%)	At the time of final follow-up (%)
Soccer	45	35	24	13
Tennis	22	18	15	13
Cycling	12	11	21	30
Running	11	11	11	18
Downhill skiing	10	0	9	8
Fitness	9	9	24	27
Squash	8	9	1	0
Gymnastics	6	2	1	0
Basketball	6	4	4	3
Swimming	6	7	6	9
Athletics	5	7	4	0
Aerobics	4	4	0	1
Volleyball	5	4	3	3
Triathlon	3	4	0	0
Ice skating	3	4	1	3

In our population, achievement of overall satisfaction with the operation was reported to be 65 % and improvement of symptoms more than 73 % (Table 4). The median NRS for pain at rest was 1 (range 0–8), that for pain when walking was 2 (range 0–9) and the NRS for pain during running was 4 (range 0–10). The subscale quality of life of the FAOS with a median of 56.3 (range 0–100) was the lowest subscale and with a median of 100 (range 99–100) was ADL highest subscale (Table 4). Seventy-five per cent of the patients (*n* = 70) did not have a re-operation, while 4 % (*n* = 4) had another arthroscopy for the OCD, one patient (1 %) had an ankle fusion, and 13 % had a hyaluronic acid injection (*n* = 12). Baseline variables were not significantly associated with the secondary outcomes.

**Table 4:** Secondary outcome measures

FAOS, median (range)		Satisfaction, *n* (%)		Change in complaints, *n* (%)	
Symptoms	64.3 (14–100)	Excellent	18 (20 %)	Completely restored	11 (12 %)
Pain	77.8 (25–100)	Good	42 (45 %)	Significantly improved	39 (42 %)
ADL	100 (99–100)	Fair	19 (20 %)	Little improved	16 (17 %)
Sport	65 (0–100)	Average	9 (10 %)	Unchanged	13 (14 %)
QOL	56.3 (0–100)	Poor	4 (4 %)	Some deteriorated	5 (5 %)
				Significantly deteriorated	5 (5 %)
				Extremely deteriorated	1 (1 %)

## Discussion

Seventy-six per cent of the patients continuing participating in sports at long-term follow-up after arthroscopic debridement and bone marrow stimulation of OCD of the talus were the most important finding of our study. However, the activity level decreased and never reached the level of that before injury. No differences were found when comparing different follow-up groups regarding the number of patients participating in sports and the activity level.

Comparing to the existing literature, Saxena and Eakin found a return to activity (high demand) rate of 95 % in approximately 17 weeks after surgery. They prospectively followed 44 patients that were divided into bone grafting and microfracturing (combined arthroscopically and open by medial malleolar osteotomy) with an average follow-up of 32 months [10].

Seijas et al. [11] treated 16 professional soccer players with an osteochondral talar defect by means of arthroscopic microfracturing. Their athletes returned to sporting activity in approximately 20 weeks, and more than 90 % of the patients were able to resume to their former sporting level after an average of 3.5 years.

Lee et al. prospectively followed 35 patients with a mean follow-up of almost 3 years (33 months). Before operation, 18 patients were actively involved in sporting activities of different intensities (basketball, baseball, tennis, wrestling), while the other 17 were interested at recreational level of sports. At follow-up, 63 % of the patient achieved their pre-injury sports level which is comparable to the results in our study [4]. The resumption of sports is lower in our study compared to the literature mentioned above. We included, however, not only athletes, but all patients participating in sports before injury. We thereby defined the activity level of patients with a validated activity level score (AAS) and were able to compare the score before injury to final follow-up.

Compared with patients treated with osteochondral transplantation, the results of our study are comparable. Paul et al. showed, however, in a retrospective design a decrease of only 8 % in the number of patients sporting compared to lifetime sports. Comparable to our study, the intensity of sports decreased as well measured in activity scales such as the Tegner and activity rating scale. They also noticed a change of sports towards lower-impact sports, such as cycling and swimming, which we observed as well [6].

Although not prospectively designed, it is shown to be the largest group of patients with the longest follow-up investigating the return to sports after arthroscopic debridement and bone marrow stimulation of talar OCDs. Besides, our study included patients that were participating in sports at any level and not only professional athletes.

The retrospective design, recall bias and low response rate were identified as limitations of our study. Because of the retrospective design, it was not possible to compare the secondary outcome to pre-operative data and have no information over the specific time to return to sports. The long-term follow-up allowed us to compare different follow-up groups and showed that the AAS as well as the number of patients that resumed sports did not deteriorate over time. Another limitation is the recall bias of the patients, which can influence the results of our study. Thereby, the study had a relatively low response rate. Possible selection bias could affect the study. Amongst the 217 patients operated in the study period were patients participating in sports as well as patients not participating in sports before injury. Before sending the questionnaire and inclusion of the patients, this was not distinguished. Therefore, the response rate stated in this study will probably be underreported.

## Conclusion

It is shown that 76 % of the patients were able to return to sports at long-term follow-up after arthroscopic debridement and bone marrow stimulation of talar OCDs. However, the activity level decreased at long-term follow-up and never reached the level of that before injury. No differences were found when comparing different follow-up groups regarding the number of patients participating in sports and the activity level. The top cited sports did not change; however, we noticed a modification towards lower-impact sports. The data of our study can be important to inform future patients on expectations after debridement and bone marrow stimulation of a talar OCD.
